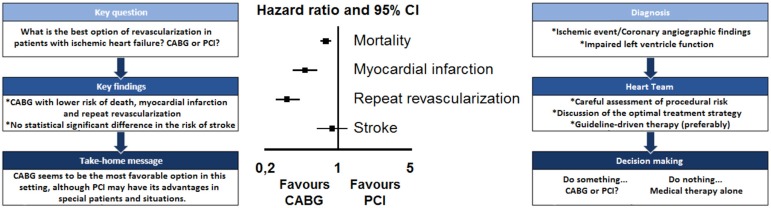# Coronary Artery Bypass Graft Surgery Improves Survival Without Increasing the Risk of Stroke in Patients with Ischemic Heart Failure in Comparison to Percutaneous Coronary Intervention: A Meta-Analysis With 54,173 Patients

**DOI:** 10.21470/1678-9741-2019-0170

**Published:** 2019

**Authors:** Michel Pompeu Barros Oliveira Sá, Álvaro Monteiro Perazzo, Felipe Augusto Santos Saragiotto, Luiz Rafael Pereira Cavalcanti, Antônio Carlos Escorel Almeida Neto, Jéssica Cordeiro Siqueira Campos, Paulo Guilherme Bezerra Braga, Sérgio da Costa Rayol, Roberto Gouvea Silva Diniz, Frederico Browne Correia Araújo Sá, Ricardo Carvalho Lima

**Affiliations:** 1 Division of Cardiovascular Surgery, Pronto Socorro Cardiológico de Pernambuco - PROCAPE, Recife, Pernambuco, Brazil.; 2 University of Pernambuco - UPE, Recife, Pernambuco, Brazil.; 3 Nucleus of Postgraduate and Research in Health Sciences, Faculty of Medical Sciences and Biological Sciences Institute - FCM/ICB, Recife, Pernambuco, Brazil.

**Keywords:** Meta-Analysis, Coronary Artery Bypass, Stents, Percutaneous Coronary Intervention, Heart Failure

## Abstract

**Objective:**

To evaluate whether there is any difference on the results of patients treated with coronary artery bypass grafting (CABG) or percutaneous coronary intervention (PCI) in the setting of ischemic heart failure (HF).

**Methods:**

Databases (MEDLINE, Embase, Cochrane Controlled Trials Register [CENTRAL/CCTR], ClinicalTrials.gov, Scientific Electronic Library Online [SciELO], Literatura Latino-americana e do Caribe em Ciências da Saúde [LILACS], and Google Scholar) were searched for studies published until February 2019. Main outcomes of interest were mortality, myocardial infarction, repeat revascularization, and stroke.

**Results:**

The search yielded 5,775 studies for inclusion. Of these, 20 articles were analyzed, and their data were extracted. The total number of patients included was 54,173, and those underwent CABG (N=29,075) or PCI (N=25098). The hazard ratios (HRs) for mortality (HR 0.763; 95% confidence interval [CI] 0.678-0.859; *P*<0.001), myocardial infarction (HR 0.481; 95% CI 0.365-0.633; *P*<0.001), and repeat revascularization (HR 0.321; 95% CI 0.241-0.428; *P*<0.001) were lower in the CABG group than in the PCI group. The HR for stroke showed no statistically significant difference between the groups (random effect model: HR 0.879; 95% CI 0.625-1.237; *P*=0.459).

**Conclusion:**

This meta-analysis found that CABG surgery remains the best option for patients with ischemic HF, without increase in the risk of stroke.

**Table:** 

Abbreviations, acronyms & symbols				
CABG	= Coronary artery bypass grafting		LVEF	= Left ventricular ejection fraction
CENTRAL/CCTR	= Cochrane Controlled Trials Register		MeSH	= Medical subject headings
CI	= Confidence interval		MI	= Myocardial infarction
EACTS	= European Association for Cardio-Thoracic Surgery		PCI	= Percutaneous coronary intervention
EF	= Ejection fraction		PICOS	= Population, Intervention, Comparison, Outcome and Study Design
ESC	= European Society of Cardiology		PRISMA	= Preferred Reporting Items for Systematic Reviews and Meta-Analyses
HF	= Heart failure		SciELO	= Scientific Electronic Library Online
HR	= Hazard ratio			
LILACS	= Literatura Latino-americana e do Caribe em Ciências da Saúde			

## INTRODUCTION

### Rationale

Recent European Society of Cardiology (ESC) and European Association for Cardio-Thoracic Surgery (EACTS) guidelines on myocardial revascularization^[1]^ clearly recommended coronary artery bypass grafting (CABG) as the first choice of revascularization strategy in patients with multivessel disease and acceptable surgical risk to improve prognosis in this scenario of left ventricular dysfunction.

According to guidelines from the United States of America^[2,3]^, revascularization strategies might be beneficial in the context of left ventricular dysfunction. CABG surgery would be class of recommendation IIa for those with moderate left ventricular dysfunction and IIb for those with left ventricular ejection fraction (LVEF) ≤35% without significant left main coronary artery disease. There is not enough data about the percutaneous coronary intervention (PCI) to allow the panels to reach any conclusion nor make any recommendation in this setting. Nevertheless, some studies^[4,5]^ have suggested that PCI could provide comparable outcomes to CABG in patients with heart failure (HF). In light of these studies, we decided to perform a systematic review with meta-analysis in order to evaluate comparatively the impact of CABG and PCI on the rates of complications and mortality of patients with ischemic HF.

### Objectives

We aimed to investigate whether there is any difference on the results of patients treated with CABG or PCI in the setting of ischemic HF. This analysis was planned in accordance with current guidelines for performing comprehensive systematic reviews and meta-analysis, including the Preferred Reporting Items for Systematic Reviews and Meta-Analyses (PRISMA)^[6]^ guidelines.

## METHODS

### Eligibility Criteria

Using Population, Intervention, Comparison, Outcome and Study Design (PICOS) strategy, studies were considered eligible if: (1) the population comprised patients with ischemic HF with impaired ejection fraction (EF); (2) there was compared efficacy between CABG and PCI; (3) the studied outcomes have included death, myocardial infarction (MI), stroke, or repeat revascularization; (4) there was a follow-up of at least 12 months. There was no restriction on language and the studies were of any type (retrospective/prospective, randomized or non-randomized, multicentric or not).

### Information Sources

The following databases were used (until February 2019): MEDLINE, Embase, the Cochrane Controlled Trials Register (CENTRAL/CCTR), ClinicalTrials.gov, the Scientific Electronic Library Online (SciELO), Literatura Latino-americana e do Caribe em Ciências da Saúde (LILACS), Google Scholar, and reference lists of relevant articles.

### Search

The following terms according to the medical subject headings (MeSH) terms included revascularization, impaired ejection fraction, LVEF, severe left ventricular dysfunction, reduced ejection fraction, heart failure, ischemic cardiomyopathy, percutaneous coronary intervention, and coronary artery bypass grafting surgery.

### Study Selection

The following steps were taken: 1) identification of titles of records through databases searching; 2) removal of duplicates; 3) screening and selection of abstracts; 4) assessment for eligibility through full-text articles; and 5) final inclusion in study. One reviewer followed steps 1 to 3. Two independent reviewers followed step 4 and selected studies. Inclusion or exclusion of studies was decided unanimously. When there was disagreement, a third reviewer made the final decision.

### Data Items

The crude endpoints were mortality, MI, stroke, and repeat revascularization.

### Data Collection Process

Two independent reviewers extracted the data. When there was disagreement about the data, a third reviewer checked them and made the final decision. From each study, we extracted patients’ characteristics, study design, and outcomes. When the data were not clearly available in the articles, we contacted the authors of the original articles by e-mail.

### Summary Measures

The principal summary measures were hazard ratio (HR) with 95% confidence interval (CI) and *P*-values (considered statistically significant when *P*<0.05) for mortality and difference in means for the other outcomes. The meta-analysis was completed with the Comprehensive Meta-Analysis software (version 2, Biostat, Inc., Englewood, New Jersey).

### Synthesis of Results

Forest plots were generated for graphical presentations of clinical outcomes, and we performed I^2^ test and χ^2^ test for the assessment of heterogeneity across the studies^[7]^. Inter-study heterogeneity was explored using the χ^2^ statistic, but the I^2^-value was calculated to quantify the degree of heterogeneity across the studies that could not be attributable to chance alone. When I^2^ was more than 50%, significant statistical heterogeneity was considered to be present. Each study was summarized by the HR, whose values were combined across the studies using a weighted DerSimonian-Laird random effects model^[8]^.

### Risk of Bias Across Studies

To assess publication bias, a funnel plot was generated for each outcome, statistically assessed by Begg and Mazumdar’s test^[9]^ and Egger’s test^[10]^.

### Sensitivity Analysis

We also investigated the influence of each study on the overall effect - by sequentially removing one study - in order to test the robustness of the main results, so that we could verify whether any study had an excessive influence on the overall results.

## RESULTS

### Study Selection

**Fig. 1:**
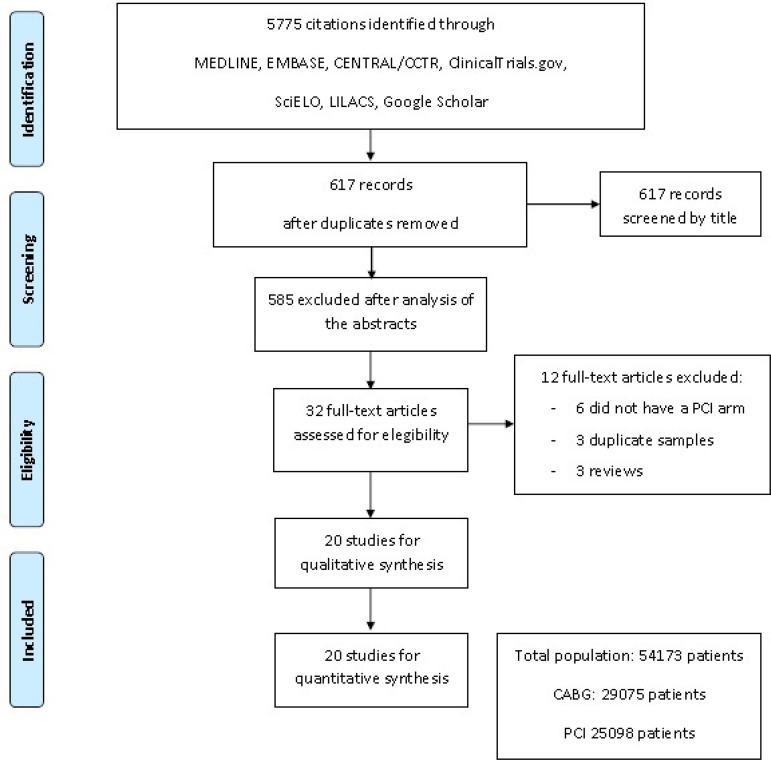
Flow diagram of studies included in data search. CABG=coronary artery bypass grafting; CENTRAL/CCTR=Cochrane Controlled Trials Register; LILACS=Literatura Latino-americana e do Caribe em Ciências da Saúde; PCI=percutaneous coronary intervention; SciELO=Scientific Electronic Library Online

A total of 5,775 citations were identified, of which 32 studies were potentially relevant and retrieved as full text. Twenty publications^[11-28]^ fulfilled our eligibility criteria. Interobserver reliability of study relevance was very good (Kappa=0.82). Agreement for decisions related to study validity was very good (Kappa=0.84). The search strategy can be seen in Figure 1.

### Study Characteristics

A total of 54,173 patients (CABG: 29,075 patients; PCI: 25,098 patients) were included, from studies published from 2002 to 2019. The studies consisted of patients whose mean age was around 65 years. Most of the patients were male in all the studies. Only two studies were randomized, seven studies were prospective, and almost all of them were multicentric. Almost all the studies had patients with LVEF <35%.

### Synthesis of Results

**Fig. 2:**
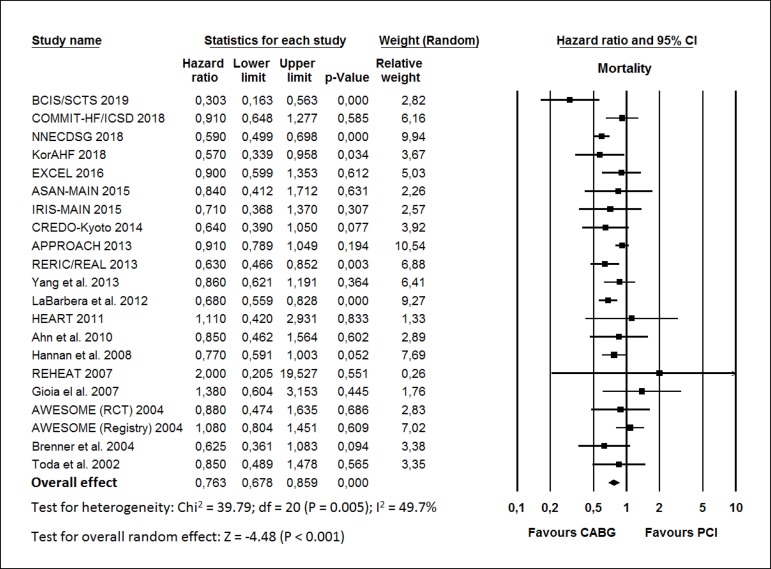
Hazard ratio and conclusions plot of mortality. CABG=coronary artery bypass grafting; CI=confidence interval; PCI=percutaneous coronary intervention

The HR for mortality in the CABG group compared with that in the PCI group in each study is reported in Figure 2. There was evidence of moderate heterogeneity of treatment effect among the studies for mortality. The overall HR (95% CI) of mortality showed better results in the CABG group (random effect model: HR 0.763; 95% CI 0.678-0.859; *P*<0.001) than in the PCI group.

**Fig. 3:**
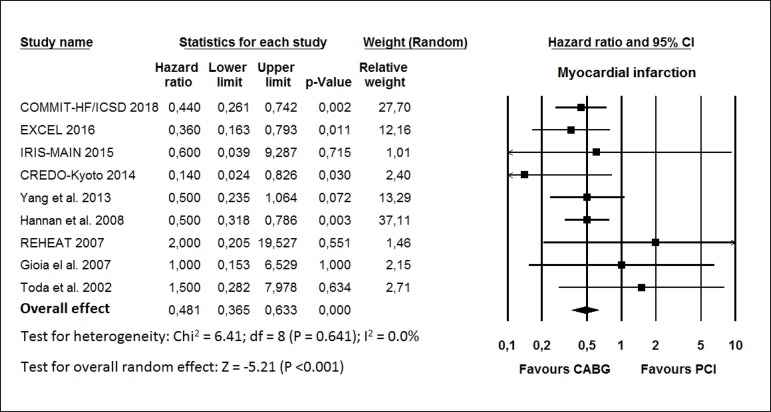
Hazard ratio and conclusions plot of myocardial infarction. CABG=coronary artery bypass grafting; CI=confidence interval; PCI=percutaneous coronary intervention

The HR for MI in the CABG group compared with that in the PCI group in each study is reported in Figure 3. There was evidence of low heterogeneity of treatment effect among the studies for MI. The overall HR (95% CI) of MI showed better results in the CABG group (random effect model: HR 0.481; 95% CI 0.365-0.633; *P*<0.001) than in the PCI group.

**Fig. 4:**
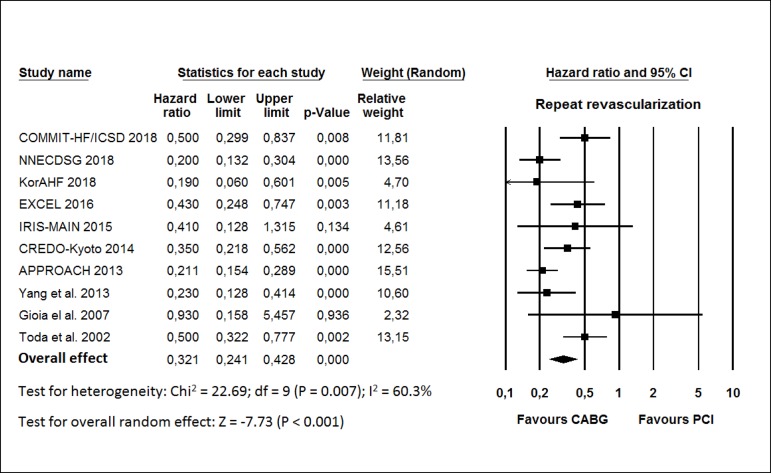
Hazard ratio and conclusions plot of repeat revascularization. CABG=coronary artery bypass grafting; CI=confidence interval; PCI=percutaneous coronary intervention

The HR for repeat revascularization in the CABG group compared with that in the PCI group in each study is reported in Figure 4. There was evidence of important heterogeneity of treatment effect among the studies for repeat revascularization. The overall HR (95% CI) of repeat revascularization showed better results in the CABG group (random effect model: HR 0.321; 95% CI 0.241-0.428; *P*<0.001) than in the PCI group.

**Fig. 5:**
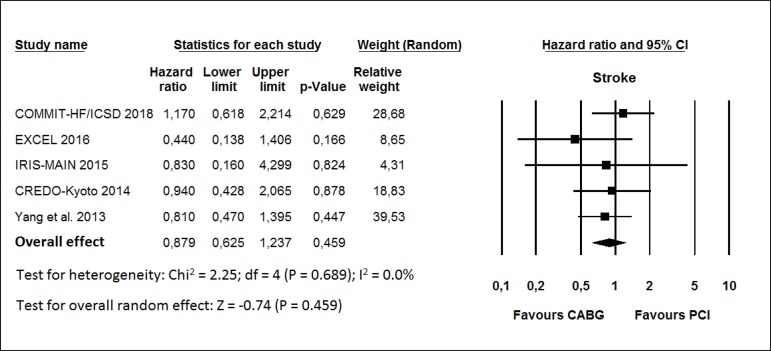
Hazard ratio and conclusions plot of stroke. CABG=coronary artery bypass grafting; CI=confidence interval; PCI=percutaneous coronary intervention

The HR for stroke in the CABG group compared with that in the PCI group in each study is reported in Figure 5. There was evidence of low heterogeneity of treatment effect among the studies for stroke. The overall HR (95% CI) of stroke showed no statistically significant difference between the groups (random effect model: HR 0.879; 95% CI 0.625-1.237; *P*=0.459).

### Risk of Bias Across Studies

**Fig. 6:**
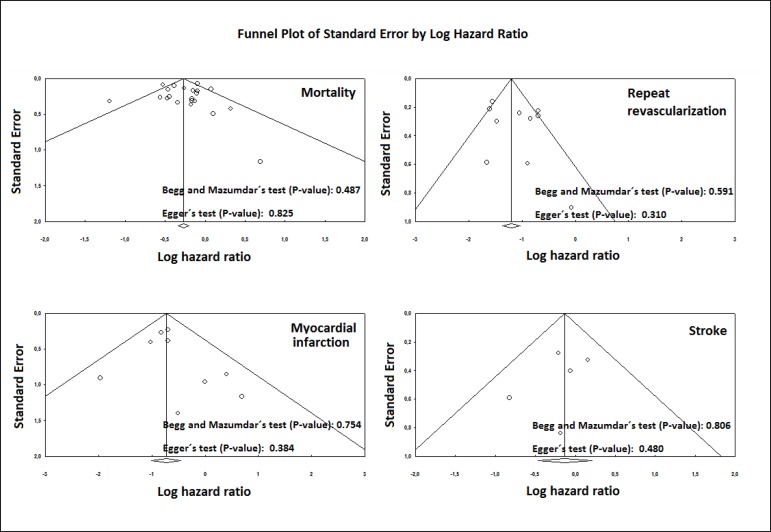
Publication bias.

Funnel plot analysis (Figure 6) disclosed no asymmetry around the axis for the outcomes, which means that there is low risk of publication bias related to these outcomes.

### Sensitivity Analysis

**Fig. 7:**
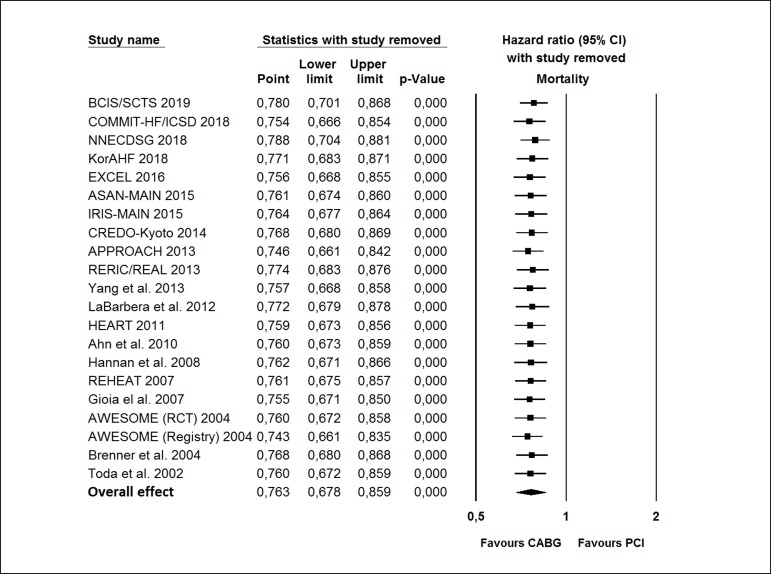
Sensitivity analysis. CABG=coronary artery bypass grafting; CI=confidence interval; PCI=percutaneous coronary intervention

Sensitivity analyses performed by removing each single study from the meta-analysis (in order to determine the influence of individual data sets on the pooled HRs) showed that none of the studies had a particular impact on the summary results of mortality (see Figure 7).

## DISCUSSION

### Summary of Evidence

To the best of our knowledge, this is the largest meta-analysis of studies performed to date that provides additional value by demonstrating that patients with ischemic HF who underwent CABG surgery have lower risk of mortality, MI, and repeat revascularization in comparison to those who underwent PCI. CABG did not increase the risk of stroke in comparison to PCI.

### What Is the Biggest Novelty of This Meta-Analysis?

Our study stands out from the crowd in that it showed no incremental risk of stroke in the CABG group in comparison with PCI in the setting of patients with HF. Several studies have suggested that CABG *vs*. PCI is associated with a significant increase of procedural stroke^[29]^, a devastating outcome with substantial mortality, morbidity, and reduced quality of life. To this date, there is a lack of conclusive evidence on the exact incidence and consequences of stroke following either CABG or PCI because individual randomized trials lacked sufficient power to detect small but meaningful differences between CABG and PCI^[30]^. Beyond mortality, it is important to consider endpoints that significantly impact quality of life, including stroke. The best evidence currently available is a patient-level meta-analysis published by Head et al.^[31]^, including 11 randomized clinical trials comparing CABG with PCI using stents. The analysis included 11,518 patients randomly assigned to PCI (N=5,753) or CABG (N=5,765) with a mean follow-up of 3.8±1.4 years. This individual patient-data pooled analysis demonstrates that 5-year stroke rates are significantly lower after PCI compared with CABG, driven by a reduced risk of stroke in the 30-day post-procedural period, but with a similar risk of stroke between 31 days and 5 years. The greater risk of stroke after CABG compared with PCI was confined to patients with multivessel disease and diabetes. Five-year mortality was markedly higher for patients experiencing a stroke within 30 days after revascularization. Our study has an almost fourfold increase in sample size, which increases the power in our study to show a significant difference if there is one. Therefore, we do not confirm this increase in the risk of stroke in the setting of patients with HF.

### Risk of Bias and Study Limitations

There are inherent limitations with meta-analyses, including the use of cumulative data from summary estimates. Patient data were gathered from published data, not from individual patient follow-up. Access to individual patient data would have enabled us to conduct further subgroup analysis and propensity analysis to account for differences between the treatment groups. This meta-analysis included data from studies that reflect the “real world” but, on the other hand, are less limited by publication bias, treatment bias, confounders, and a certain tendency to overestimate treatment effects observed in the observational studies, since patient selection alters the outcome and, thus, makes non-randomized studies less robust.

Moreover, considerable statistical heterogeneity was observed in some analyses, but we used the random-effects model to counterbalance this aspect. We also observed low risk of publication bias in the outcomes. We must remind the readers of the fact that a research with statistically significant results is more likely to be submitted to medical journals and published than a work with null or non-significant results, being the former also more likely to appear more prominently in English, in higher impact journals. All the aforementioned aspects lead to the appearance of publication biases, but, in this case, we cannot state that the impact of CABG in comparison to PCI on morbidity and mortality rates observed in our study is solely due to bias.

## CONCLUSION

This meta-analysis found that CABG surgery remains the best option for patients with ischemic HF.

**Table:** 

Authors' roles & responsibilities	
MPBOS	Substantial contributions to the conception or design of the work; or the acquisition, analysis, or interpretation of data for the work; agreement to be accountable for all aspects of the work in ensuring that questions related to the accuracy or integrity of any part of the work are appropriately investigated and resolved; final approval of the version to be published
AMP	Drafting the work or revising it critically for important intellectual content; agreement to be accountable for all aspects of the work in ensuring that questions related to the accuracy or integrity of any part of the work are appropriately investigated and resolved; final approval of the version to be published
FASS	Drafting the work or revising it critically for important intellectual content; final approval of the version to be published
LRPC	Drafting the work or revising it critically for important intellectual content; agreement to be accountable for all aspects of the work in ensuring that questions related to the accuracy or integrity of any part of the work are appropriately investigated and resolved; final approval of the version to be published
ACEAN	Drafting the work or revising it critically for important intellectual content; final approval of the version to be published
JCSC	Drafting the work or revising it critically for important intellectual content; final approval of the version to be published
PGBB	Drafting the work or revising it critically for important intellectual content; final approval of the version to be published
SCR	Drafting the work or revising it critically for important intellectual content; final approval of the version to be published
RGSD	Drafting the work or revising it critically for important intellectual content; final approval of the version to be published
FBCAS	Drafting the work or revising it critically for important intellectual content; agreement to be accountable for all aspects of the work in ensuring that questions related to the accuracy or integrity of any part of the work are appropriately investigated and resolved; final approval of the version to be published
RCL	Drafting the work or revising it critically for important intellectual content; final approval of the version to be published

## Figures and Tables

**Figure:**